# Selective Effects of the Loss of NMDA or mGluR5 Receptors in the Reward System on Adaptive Decision-Making

**DOI:** 10.1523/ENEURO.0331-18.2018

**Published:** 2018-10-05

**Authors:** Przemysław Eligiusz Cieślak, Woo-Young Ahn, Rafał Bogacz, Jan Rodriguez Parkitna

**Affiliations:** 1 Department of Molecular Neuropharmacology, Institute of Pharmacology of the Polish Academy of Sciences, 31-343, Krakow, Poland; 2 Department of Psychology, Seoul National University, Seoul 08826, Korea; 3 MRC Brain Networks Dynamics Unit, Nuffield Department of Clinical Neurosciences, Oxford University, John Radcliffe Hospital, Oxford OX3 9DU, United Kingdom

**Keywords:** decision-making, dopamine, glutamate receptors, mouse behavior, reinforcement learning

## Abstract

Selecting the most advantageous actions in a changing environment is a central feature of adaptive behavior. The midbrain dopamine (DA) neurons along with the major targets of their projections, including dopaminoceptive neurons in the frontal cortex and basal ganglia, play a key role in this process. Here, we investigate the consequences of a selective genetic disruption of NMDA receptor and metabotropic glutamate receptor 5 (mGluR5) in the DA system on adaptive choice behavior in mice. We tested the effects of the mutation on performance in the probabilistic reinforcement learning and probability-discounting tasks. In case of the probabilistic choice, both the loss of NMDA receptors in dopaminergic neurons or the loss mGluR5 receptors in D_1_ receptor-expressing dopaminoceptive neurons reduced the probability of selecting the more rewarded alternative and lowered the likelihood of returning to the previously rewarded alternative (win-stay). When observed behavior was fitted to reinforcement learning models, we found that these two mutations were associated with a reduced effect of the expected outcome on choice (i.e., more random choices). None of the mutations affected probability discounting, which indicates that all animals had a normal ability to assess probability. However, in both behavioral tasks animals with targeted loss of NMDA receptors in dopaminergic neurons or mGluR5 receptors in D_1_ neurons were significantly slower to perform choices. In conclusion, these results show that glutamate receptor-dependent signaling in the DA system is essential for the speed and accuracy of choices, but at the same time probably is not critical for correct estimation of probable outcomes.

## Visual Abstract

**Figure:**
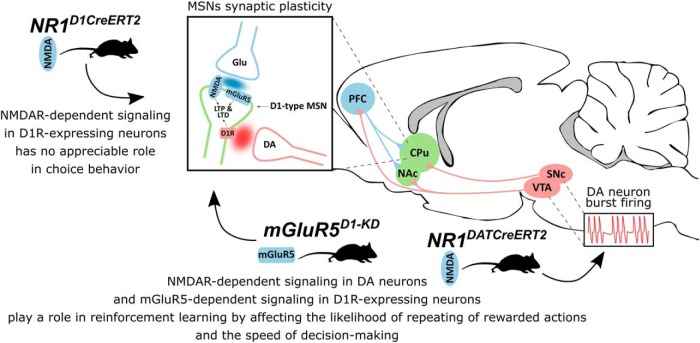

## Significance Statement

We investigated the role of glutamate signaling in the reward system of the brain in adaptive decision-making. We used genetically modified mice with a disruption of glutamate signaling that was caused by the deletion of glutamate receptors in dopamine-producing and dopamine-sensitive neurons. When mutant mice were offered a choice between two alternatives with varying chances of being rewarded, the mutations decreased the probability of selecting the more often rewarded alternative, and the likelihood of repeating a previously rewarded choice. Moreover, mutant animals were much slower in performing choices. Our results show that when glutamate signaling in the reward system is disrupted, it causes an impairment in decision-making by increasing randomness and reducing the speed of the decision-making process.

## Introduction

Midbrain dopamine (DA) neurons originate from the ventral tegmental area and substantia nigra and, along with the major targets of their projections, including dopaminoceptive neurons in the frontal cortex and basal ganglia, play a central role in the organization of adaptive behavior (Berridge and Robinson, 1998; Wise, 2004; Floresco and Magyar, 2006; Salamone and Correa, 2012). In rodents and nonhuman primates, the burst firing of midbrain DA neurons and the subsequent phasic release of DA encode reward prediction error (Schultz et al., 1997; Bayer and Glimcher, 2005; Hart et al., 2014). This error in reward expectation is a signal of the need to modify synaptic plasticity at corticostriatal synapses and update the action values stored by striatal neurons (Reynolds et al., 2001; Samejima et al., 2005; Lee et al., 2012). In this way, the DA system provides a neural substrate for reinforcement learning mechanisms underlying decision-making and action selection (Glimcher, 2011; Schultz, 2015). It should be noted though that the role of DA extends beyond reinforcement learning, as it is also involved in the regulation of motivation and vigor as well as the performance of instrumental behavior (Salamone and Correa, 2012; Shiner et al., 2012).

The activity and plasticity in the DA system are largely dependent on excitatory glutamatergic transmission. Glutamatergic inputs activate NMDA receptors and drive the burst firing in DA neurons (Overton and Clark, 1992; Chergui et al., 1993), phasic DA release (Sombers et al., 2009; Wickham et al., 2013), and induction of long-term potentiation onto the dopaminergic neurons underlying cue–reward learning (Stuber et al., 2008; Harnett et al., 2009). Moreover, NMDA receptors and metabotropic glutamate receptor 5 (mGluR5) are crucial for the induction of synaptic and structural plasticity in dopaminoceptive striatal medium spiny neurons (Calabresi et al., 2007; Shen et al., 2008; Surmeier et al., 2009; Yagishita et al., 2014). Altogether, these observations indicate that glutamate-dependent signaling is crucial for DA-mediated reinforcement. However, in most studies, the observations are based on correlations and *in vitro* measurements; therefore, the causality or degree of contribution remains uncertain.

A more direct approach for testing the role of glutamate-dependent signaling in reinforcement learning is the use of genetically modified mice with an inactivation of glutamate receptors in DA or dopaminoceptive neurons. Such models have been generated and generally observed to result in impairments in tasks involving instrumental and pavlovian learning, confirming that a disruption in glutamate-dependent signaling in the DA system is sufficient to cause an impairment in reward-based learning (Zweifel et al., 2009; Novak et al., 2010; Parker et al., 2010, 2011; Beutler et al., 2011; Wang et al., 2011; James et al., 2015). However, most experiments were conducted using paradigms in which only a single lever or conditioned stimulus was reinforced. Therefore, a crucial aspect of adaptive decision-making (i.e., choosing among competing courses of action in a changing environment) was not comprehensively addressed in those studies.

Here, we sought to determine the contribution of glutamate receptor-dependent signaling in DA and dopaminoceptive neurons to adaptive decision-making. We used mice with cell type-specific, tamoxifen-inducible inactivation of NMDA receptors in DA and D_1_ receptor-expressing neurons (Engblom et al., 2008; Jastrzębska et al., 2016; Sikora et al., 2016) and animals with a knockdown of mGluR5 receptors in D_1_ neurons (Novak et al., 2010; Rodriguez Parkitna et al., 2013). The animals were tested using a probabilistic reinforcement learning task, in which the mouse is required to estimate the expected value of two alternatives associated with different reward probabilities by trial and error. This task was followed by a probability-discounting task in which the animal is required to choose between two options that provide rewards that differ in magnitude (small vs large) and probability (certain vs uncertain).

## Materials and Methods

### Animals

The following three strains of genetically modified mice were used in the study: NR1^DATCreERT2^ mice, which had an inducible deletion of the NR1 subunit of the NMDA receptor in DA transporter (DAT)-expressing neurons (Engblom et al., 2008; Jastrzębska et al., 2016); NR1^D1CreERT2^ animals, which had an inducible loss of the NR1 subunit of the NMDA receptor in D_1_ receptor-expressing neurons (Sikora et al., 2016); and mGluR5^KD-D1^ mice, which had a selective knockdown of the mGluR5 receptor in D_1_-expressing neurons (Novak et al., 2010; Rodriguez Parkitna et al., 2013). All strains were bred to be congenic with the C57BL/6N strain. Genotyping was performed as previously described. The animals were housed two to five animals per cage in a room with a controlled temperature at 22 ± 2°C under a 12 h light/dark cycle. Unless otherwise indicated, the mice had *ad libitum* access to tap water and standard rodent laboratory chow.

Regarding the CreERT2-dependent mutations, the recombination was induced in adult animals at the age of 8–10 weeks using tamoxifen treatment. Tamoxifen (Sigma-Aldrich) was dissolved in sunflower oil, filtered through a 0.22 μm membrane, and injected intraperitoneally once a day for 5 consecutive days at a dose of 100 mg/kg and a volume of 5 μl/g. The genotype of the mutant mice was [Tg/0; flox/flox], and the genotype of the control animals was [0/0; flox/flox]. All tamoxifen-treated animals were allowed to rest for at least 3 weeks before the start of the behavioral procedures. Regarding mGluR5^KD-D1^, no induction was necessary, and the expression of the transgene was initiated when the D_1_ promoter became active during late development. The genotype of the mutant mGluR5^KD-D1^ animals was [Tg/0], and the genotype of their respective controls was [0/0].

Only male mice were used in the study. The mean ages and weights of the cohorts of animals used in the experiments were as follows: 16.25 ± 1.05 weeks and 25.6 ± 0.85 × *g* for the NR1^DATCreERT2^ mice and 16.57 ± 1.15 weeks and 29.43 ± 0.62 × *g* for their respective controls; 18.33 ± 0.94 weeks and 26.39 ± 1.21 × *g* for the NR1^D1CreERT2^ mice and 19.33 ± 1.08 weeks and 27.85 ± 1.37 × *g* for their controls; and 13.38 ± 1.31 weeks and 25.8 ± 1.1 × *g* for the mGluR5^KD-D1^ mice and 13.56 ± 1.12 weeks and 24.98 ± 1.02 × *g* for their controls. The same cohorts of animals were used in the probabilistic reinforcement learning and probability-discounting tasks.

### Behavioral procedures

#### Water deprivation

A week before the behavioral testing, water consumption was limited to 1–1.5 ml/d, and this water restriction schedule was maintained for the duration of the experiments. The mice were trained 5–7 d/week, and their body weight was monitored daily. The water restriction was lessened if the mice fell to <85% of their body weight from the beginning of the deprivation.

#### Apparatus

The experiments were performed using mouse operant chambers (ENV-307W-CT, Med Associates) enclosed in cubicles that were equipped with a fan to provide ventilation and mask extraneous noise. Each chamber was equipped with a dual cup liquid receptacle, a nose-poke port containing a cue light located on each side of a liquid receptacle, and a house light located on the wall opposite to the liquid receptacle. Saccharin-flavored water (0.01% w/v saccharin; Sigma-Aldrich) was delivered into an individual cup by an infusion pump (PHM-100, Med Associates) connected to the liquid receptacle via a silicone tube. The amount of fluid delivered (reward size) was dependent on the duration of the infusion.

#### Training

First, the mice were placed in the operant chamber for 30 min, during which 20 μl of water were delivered into the receptacle in 60 s intervals. This procedure allowed the animals to become familiar with the chamber and liquid reward. On subsequent days, the mice were trained under a continuous reinforcement schedule and were rewarded with 10 μl of water after poking their noses into the active port (with the cue-light on). The other port was inactive. The nose pokes in the inactive port were recorded but had no consequences. The port assignment was counterbalanced, and the animals were trained until they reached the criterion of 60 rewarded responses in 40 min, which occurred first in one port and then in the other port in a subsequent session. This training was followed by additional training during which the left and right ports were active once in every pair of trials, and the order within the pair was random. These sessions ended when an animal completed 100 trials or 60 min elapsed, whichever came first. There was no limit to the trial duration, and each trial ended when a nose poke in the active port resulted in the delivery of a reward, followed by a 5 s intertrial interval (ITI). The animals had to complete at least 85 trials. Finally, the mice underwent omission training, which was similar to the training described above with two exceptions. First, the trial number was increased to 160. Second, responding in an active poke resulted in a 50% chance of reward omission. Reward omission was signaled by switching on the house light for the duration of the ITI. The animals had to complete at least 120 trials.

#### Probabilistic reinforcement learning task

**Figure 1.**
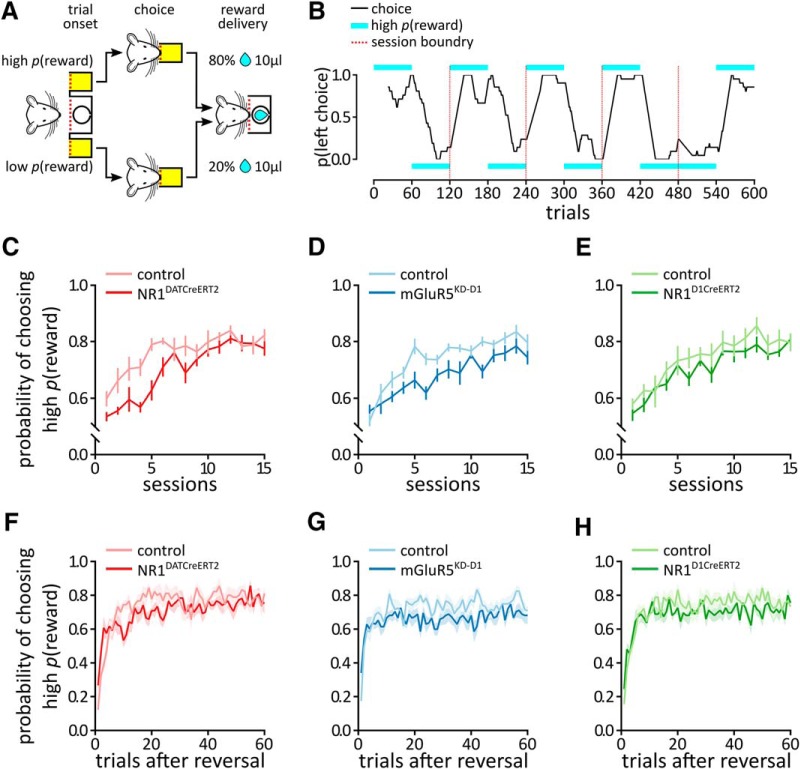
The probabilistic reinforcement learning task. *A*, Schematic representation of the probabilistic reinforcement learning task. The animal could make a nose-poke in one of two ports. Following a nose-poke, water could have been delivered with the probability depending on the chosen port. The nose-poke ports were randomly assigned 80% or 20% reward probabilities. During each session, the reward probabilities were reversed after 60 trials. *B*, An example the choice behavior of a mouse in 600 trials (sessions 6–10). The black line shows the probability of choosing the left side (data smoothed with the 21 point moving average). The cyan bars indicate the side with the higher probability of reward delivery. The red dashed line indicates session boundaries. *C–H*, Probability of selecting the alternative with the higher reward probability by the NR1^DATCreERT2^ (mutant, *n* = 6; control, *n* = 8; *C*, *F*), mGluR5^KD-D1^ (mutant, *n* = 8; control, *n* = 9; *D*, *G*), and NR1^D1CreERT2^ (mutant, *n* = 6; control, *n* = 9; *E*, *H*) strains. *C–E*, Session-by-session analysis; data were collapsed across trials. *F–H*, Trial-by-trial analysis; data were collapsed across sessions. Data are represented as the mean ± SEM.

In this task, the nose-poke ports were randomly assigned reward probabilities of 80% or 20% (Fig. 1*A*). During each session, the reward probabilities were reversed after 60 trials. Thus, to maximize the long-term sum of the rewards, the mouse had to select the alternative with the higher success probability and adapt its choices to the changes in the reward contingencies. There was no limit to the trial duration, and the session ended when the animal completed 120 trials or 60 min elapsed. Rewarded choices resulted in the delivery of 10 μl of water, followed by a 5 s ITI. Unrewarded choices were signaled by turning on the house light for the duration of the ITI. The animals were trained in this task for 15 sessions.

#### Probability-discounting task

In this task, one nose-poke port was associated with the delivery of a small reward (10 μl), while the other nose-poke port was associated with the delivery of a large reward (20 μl). Each session consisted of 20 forced trials, followed by 40 free choice trials (see Fig. 5*A*). During the forced trials, only one port was active, whereas during the free choice trials, both ports were active. Once the preference for the large reward was stabilized, the probability of its delivery gradually decreased to 75%, 50%, or 25% during subsequent blocks of four to five sessions. Simultaneously, the small reward was always available at a 100% probability. The trials were separated by a 5 s ITI, and unrewarded choices were signaled by turning on the house light for the duration of the ITI.

### Statistical analysis

A script written in R was used to parse the data files that were generated during the behavioral experiments. All statistical analyses were conducted using GraphPad Prism 7 (GraphPad Software) and R software. Statistical significance was estimated using an ANOVA, followed by a Bonferroni *post hoc* test or a Student’s *t* test, as appropriate. The results were considered significant at α = 0.05. One animal from the control group in the NR1^DATCreERT2^ strain was classified as an outlier (Grubb’s test) in choice reaction time measures (in both tasks) and was excluded from all analyses. Two animals (one from the NR1^DATCreERT2^ strain and one control mouse from the NR1^D1CreERT2^ strain) showed no preference for the freely available large reward in the probability-discounting task (0.5 ± 0.5% and 1.5 ± 0.6%, respectively) and were excluded from the analysis of this task, to avoid the misinterpretation of the effect of discounting. Confidence intervals (CIs) for *post hoc* comparisons are listed in Table 1.

**Table 1.** Statistical table

Figure	Data structure	Type of test	95% CIs or 95% HDIs
Figure 2*A*_η+_	Assumed normal distribution	Hyperposterior distribution	(−0.3601, 0.0779)
Figure 2*A*_η−_	Assumed normal distribution	Hyperposterior distribution	(−0.2952, 0.129)
Figure 2*A*_β_	Assumed normal distribution	Hyperposterior distribution	(−0.461, −0.1018)
Figure 2*B*_η+_	Assumed normal distribution	Hyperposterior distribution	(−0.3081, 0.2367)
Figure 2*B*_η−_	Assumed normal distribution	Hyperposterior distribution	(−0.2532, 0.4388)
Figure 2*B*_β_	Assumed normal distribution	Hyperposterior distribution	(−0.5163, −0.1429)
Figure 2*C*_η+_	Assumed normal distribution	Hyperposterior distribution	(−0.3631, 0.0847)
Figure 2*C*_η−_	Assumed normal distribution	Hyperposterior distribution	(−0.2793, 0.2792)
Figure 2*C*_β_	Assumed normal distribution	Hyperposterior distribution	(−0.4919, 0.2115)
Figure 3*A*_win-stay_	Assumed normal distribution	Two-tailed *t* test	(−0.126, −0.03798)
Figure 3*A*_lose-shift_	Assumed normal distribution	Two-tailed *t* test	(−0.05501, 0.1081)
Figure 3*B*_win-stay_	Assumed normal distribution	Two-tailed *t* test	(−0.1675, −0.03254)
Figure 3*B*_lose-shift_	Assumed normal distribution	Two-tailed *t* test	(−0.01564, 0.1316)
Figure 3*C*_win-stay_	Assumed normal distribution	Two-tailed *t* test	(−0.1263, 0.01433)
Figure 3*C*_lose-shift_	Assumed normal distribution	Two-tailed *t* test	(−0.01022, 0.1521)
Figure 3*D*_win-stay_	Assumed normal distribution	Two-tailed *t* test	(−0.126, −0.03624)
Figure 3*D*_lose-shift_	Assumed normal distribution	Two-tailed *t* test	(−0.0746, 0.04841)
Figure 3*E*_win-stay_	Assumed normal distribution	Two-tailed *t* test	(−0.1797, −0.03847)
Figure 3*E*_lose-shift_	Assumed normal distribution	Two-tailed *t* test	(−0.02373, 0.05712)
Figure 3*F*_win-stay_	Assumed normal distribution	Two-tailed *t* test	(−0.1268, 0.01674)
Figure 3*F*_lose-shift_	Assumed normal distribution	Two-tailed *t* test	(−0.02587, 0.1276)
Figure 4*B*_lose_	Assumed normal distribution	Bonferroni-corrected *t* test	(−0.151, −9.144)
Figure 4*B*_win_	Assumed normal distribution	Bonferroni-corrected *t* test	(−5.636, −14.629)
Figure 4*B*_control_	Assumed normal distribution	Bonferroni-corrected *t* test	(0.535, −9.078)
Figure 4*B*_mutant_	Assumed normal distribution	Bonferroni-corrected *t* test	(−5.594, −13.919)
Figure 4*C*	Assumed normal distribution	Two-tailed *t* test	(0.07629, 0.2383)
Figure 4*E*_lose_	Assumed normal distribution	Bonferroni-corrected *t* test	(1.211, −5.670)
Figure 4*E*_win_	Assumed normal distribution	Bonferroni-corrected *t* test	(−2.656, −9.537)
Figure 4*E*_control_	Assumed normal distribution	Bonferroni-corrected *t* test	(−4.688, −11.769)
Figure 4*E*_mutant_	Assumed normal distribution	Bonferroni-corrected *t* test	(−8.758, −15.433)
Figure 4*F*	Assumed normal distribution	Two-tailed *t* test	(0.03127, 0.2603)
Figure 4*H*_lose_	Assumed normal distribution	Bonferroni-corrected *t* test	(3.862, −5.833)
Figure 4*H*_win_	Assumed normal distribution	Bonferroni-corrected *t* test	(2.707, −6.988)
Figure 4*H*_control_	Assumed normal distribution	Bonferroni-corrected *t* test	(1.383, −9.238)
Figure 4*H*_mutant_	Assumed normal distribution	Bonferroni-corrected *t* test	(−0.746, −9.418)
Figure 4*I*	Assumed normal distribution	Two-tailed *t* test	(−0.09834, 0.06706)
Figure 5*B*_100%_	Assumed normal distribution	Bonferroni-corrected *t* test	(44.207, −33.477)
Figure 5*B*_75%_	Assumed normal distribution	Bonferroni-corrected *t* test	(42.318, −35.366)
Figure 5*B*_50%_	Assumed normal distribution	Bonferroni-corrected *t* test	(46.199, −31.485)
Figure 5*B*_25%_	Assumed normal distribution	Bonferroni-corrected *t* test	(23.622, −54.062)
Figure 5*C*_100%_	Assumed normal distribution	Bonferroni-corrected *t* test	(18.145, −26.659)
Figure 5*C*_75%_	Assumed normal distribution	Bonferroni-corrected *t* test	(13.173, −31.631)
Figure 5*C*_50%_	Assumed normal distribution	Bonferroni-corrected *t* test	(18.416, −26.388)
Figure 5*C*_25%_	Assumed normal distribution	Bonferroni-corrected *t* test	(18.624, −26.179)
Figure 5*D*_100%_	Assumed normal distribution	Bonferroni-corrected *t* test	(25.957, −20.935)
Figure 5*D*_75%_	Assumed normal distribution	Bonferroni-corrected *t* test	(18.868, −28.024)
Figure 5*D*_50%_	Assumed normal distribution	Bonferroni-corrected *t* test	(1.613, −45.279)
Figure 5*D*_25%_	Assumed normal distribution	Bonferroni-corrected *t* test	(19.101, −27.791)
Figure 6*A* forced _100%_	Assumed normal distribution	Bonferroni-corrected *t* test	(−8.519, −16.751)
Figure 6*A* forced _75%_	Assumed normal distribution	Bonferroni-corrected *t* test	(−7.398, −15.630)
Figure 6*A* forced _50%_	Assumed normal distribution	Bonferroni-corrected *t* test	(−6.524, −14.756)
Figure 6*A* forced _25%_	Assumed normal distribution	Bonferroni-corrected *t* test	(−4.346, −12.578)
Figure 6*A* free _100%_	Assumed normal distribution	Bonferroni-corrected *t* test	(−6.166, −15.530)
Figure 6*A* free _75%_	Assumed normal distribution	Bonferroni-corrected *t* test	(−2.561, −11.925)
Figure 6*A* free _50%_	Assumed normal distribution	Bonferroni-corrected *t* test	(−1.947, −11.312)
Figure 6*A* free _25%_	Assumed normal distribution	Bonferroni-corrected *t* test	(−1.615, −10.979)
Figure 6*B* forced _100%_	Assumed normal distribution	Bonferroni-corrected *t* test	(−2.772, −8.595)
Figure 6*B* forced _75%_	Assumed normal distribution	Bonferroni-corrected *t* test	(−1.419, −7.243)
Figure 6*B* forced _50%_	Assumed normal distribution	Bonferroni-corrected *t* test	(−0.924, −6.748)
Figure 6*B* forced _25%_	Assumed normal distribution	Bonferroni-corrected *t* test	(−1.544, −7.368)
Figure 6*B* free _100%_	Assumed normal distribution	Bonferroni-corrected *t* test	(−0.135, −7.745)
Figure 6*B* free _75%_	Assumed normal distribution	Bonferroni-corrected *t* test	(0.421, −7.189)
Figure 6*B* free _50%_	Assumed normal distribution	Bonferroni-corrected *t* test	(0.512, −7.098)
Figure 6*B* free _25%_	Assumed normal distribution	Bonferroni-corrected *t* test	(0.636, −6.974)
Figure 6*C* forced _100%_	Assumed normal distribution	Bonferroni-corrected *t* test	(2.767, −5.107)
Figure 6*C* forced _75%_	Assumed normal distribution	Bonferroni-corrected *t* test	(3.120, −4.754)
Figure 6*C* forced _50%_	Assumed normal distribution	Bonferroni-corrected *t* test	(3.388, −4.486)
Figure 6*C* forced _25%_	Assumed normal distribution	Bonferroni-corrected *t* test	(2.297, −5.578)
Figure 6*C* free _100%_	Assumed normal distribution	Bonferroni-corrected *t* test	(2.447, −5.754)
Figure 6*C* free _75%_	Assumed normal distribution	Bonferroni-corrected *t* test	(3.649, −4.552)
Figure 6*C* free _50%_	Assumed normal distribution	Bonferroni-corrected *t* test	(4.181, −4.020)
Figure 6*C* free _25%_	Assumed normal distribution	Bonferroni-corrected *t* test	(0.952, −7.249)

### Computational modeling

We fitted three reinforcement learning models to trial-by-trial choice data of the probabilistic reinforcement learning task, which are all based on the Rescorla-Wagner model (Rescorla and Wagner, 1972), but include additional features. Model 1 assumes that animals learn with different rates when the reward prediction error is positive and negative (den Ouden et al., 2013). Model 2 assumes that the animals have learned that entering only one of the ports results in a high reward probability, so in this model after choosing one option, the expected rewards for both options are modified in opposite directions (Gläscher et al., 2009). Model 3 integrates models 1 and 2, so it includes separate learning rates for positive and negative prediction errors of the chosen option and updates the unchosen option using the fictitious learning component of model 2.

As model 3 is the most general, we start with its description, and then present how it can be simplified to give models 1 and 2. In model 3, the expected value of the chosen (*V_c_*_,_*_t_*) and unchosen (*V_uc_*_,_*_t_*) options are updated as follows on each trial *t*. If prediction error on trial *t* ($PE_{t}=r_{t}-V_{c,t}$) is ≥0, expected values of chosen and unchosen options are updated with learning rate $\eta_{+}({0\leq \eta }_{+}\leq 1)$, as follows:

(1) $$V_{c,t+1}=V_{c,t}+\eta_{+}\cdot \left(r_{t}-V_{c,t}\right)$$

(2) $$V_{uc,t+1}=V_{uc,t}+\eta_{+}\cdot \left(-r_{t}-V_{uc,t}\right).$$

Note that the unchosen option is updated with a fictitious prediction error $\left(PE_{t}=-r_{t}-V_{uc,t}\right)$ following the study by Gläscher et al. (2009). If $PE_{t}$ is <0, the expected values of chosen and unchosen options are updated with learning rate $\eta_{-}({0\leq \eta }_{-}\leq 1)$:

(3) $$V_{c,t+1}=V_{c,t}+\eta_{-}\cdot \left(r_{t}-V_{c,t}\right)$$

(4) $$V_{uc,t+1}=V_{uc,t}+\eta_{-}\cdot \left(-r_{t}-V_{uc,t}\right).$$

In the simulations, $r_{t}$ is set to 1 if reward is received on trial *t*, or to −1 if it is omitted. Choice probabilities are computed based on the expected values as follows. If *A* and *B* refer to the two options of the probabilistic reinforcement learning task and $p_{t+1}\left(A\right)$ refers to the probability of choosing the option A on trial *t* + 1, then:

(5) $$p_{t+1}\left(A\right)=\frac{1}{1+e^{-\beta \cdot (V_{A,t}-V_{B,t})}}.$$

Here, $\beta \left(0\leq \beta \right)$ is the inverse temperature parameter, which governs the degree of exploitation and exploration (i.e., low and high values of β indicate more exploration and exploitation, respectively). In summary, model 3 has three free parameters: $\eta_{+}$ (learning rate for positive PE), $\eta_{-}$ (learning rate for negative PE) and β (inverse temperature). If we set $\eta_{+}=\eta \_=\eta$, the model becomes model 2, which has two free parameters: η (learning rate) and β (inverse temperature). If we only update the values of chosen options using Equations 1 and 3 (but not use Equations 2 and 4), the model becomes model 1, which also has three free parameters: $\eta_{+}$ (learning rate for positive PE), $\eta_{-}$ (learning rate for negative PE), and β (inverse temperature).

We fitted the three models using hierarchical Bayesian analysis (HBA), which pools information across individuals and allows us to capture both individual differences and commonalities across subjects in a reliable way (Shiffrin et al., 2008; Ahn et al., 2011; Lee, 2011). To perform HBA, we used the hBayesDM package (Ahn et al., 2017), which is an R package that offers HBA of various computational models and tasks using the Stan software (Carpenter et al., 2017). The hBayesDM functions of models 1–3 are *prl_rp*, *prl_fictitious_woa*, and *prl_fictitious_rp_woa*, respectively. All source codes and Bayesian model formulation are available in its GitHub repository: https://github.com/CCS-Lab/hBayesDM. We performed model comparisons and identified a best-fitting model using leave-one-out cross-validation information criterion (LOOIC). To compute LOOIC for a given model we used the *loo* R package, which computes leave-one-out predictive density using Pareto smoothed importance sampling (Vehtari et al., 2017). The LOOIC inherently penalizes model complexity, as an overly complicated model will perform poorly on unseen data than a simpler model. It also has an advantage over other measures designed to prevent overfitting by overly complex model (like Akaike or Bayesian information criterion) in that it measures the overfitting directly.

### Simulation Analysis

To test whether the best-fitting model can describe the observed data well, we performed simulation analysis as previously described (Ahn et al., 2008; Steingroever et al., 2014). Briefly, by using estimated individual parameters alone (without access to trial-by-trial choice history), we generated simulated agents and computed their win-stay and lose-shift (switching to the alternative choice when the preceding response yielded no reward) probabilities. When we generated simulated data, for each group and condition, we used its total number of trials and subjects of the real data. Then, we simulated choices on the probabilistic reinforcement learning task using estimated individual parameters (individual posterior means) of each simulated agent for the whole trajectory (i.e., 1800 trials) using customized R codes.

## Results

### Performance in the probabilistic reinforcement learning task

The animals were tested in a probabilistic reinforcement learning task in which they could choose between two alternatives with either an 80% or 20% chance of being rewarded with 10 μl of water (Fig. 1*A*). The test consisted of 15 sessions, and each session consisted of 120 trials. The trials were not time limited. The initial assignment of the reward probabilities was random and reversed in the middle of each session. An example of the choice behavior of a mouse over 600 trials (sessions 6–10) is shown in Fig. 1*B*.

All groups, regardless of their genotype, showed a significant increase in the frequency of selecting the more often rewarded alternative over the course of the experiment (Fig. 1*C*: session, *F*_(14,168)_ = 17.15; Fig. 1*D*: session F_14,210_ = 20.69; Fig. 1*E*: session, *F*_(14,182)_ = 19.17; all *p* < 0.0001). The NR1^DATCreERT2^ mice chose the alternative with the higher reward probability on a smaller fraction of trials (Fig. 1*C*: genotype, *F*_(1,12)_ = 11.50, *p* = 0.0054). However, this difference was due to initial slower increase in choosing the correct option, and the mutants eventually reached the same performance levels as the control animals (genotype × session: *F*_(14,168)_ = 1.90, *p* = 0.0298). In contrast, in the mGluR5^KD-D1^ mice, the probability of choosing the alternative with the higher reward probability was consistently lower (Fig. 1*D*: genotype, *F*_(1,15)_ = 12.62, *p* = 0.0029; genotype × session, *F*_(14,210)_ = 1.49, *p* = 0.1180). The choice behavior of the NR1^D1CreERT2^ mice did not differ from that of the controls (Fig. 1*E*: genotype, *F*_(1,13)_ = 1.79, *p* = 0.2034; genotype × session, *F*_(14,182)_ = 0.53, *p* = 0.9103).

Figure 1*F–H* shows the probability of choosing the correct option in the 60 trials after reversal (average based on all sessions). The probability was initially <50%, as mice choose the option that was rewarded more frequently before reversal, but then quickly increased (Fig. 1*F*: trial, *F*_(59,708)_ = 12.6; Fig. 1*G*: trial, *F*_(59,885)_ = 7.03; Fig. 1*H*: trial, *F*_(59,767)_ = 9.67; all *p* < 0.0001). The effects of mutations in Figure 1*F*–*H* parallel those observed in Figure 1*C–E*. The NR1^DATCreERT2^ mice were initially slower in choosing the alternative with the higher reward probability, but eventually reached the same performance levels as the control animals (Fig. 1*F*: genotype, *F*_(1,12)_ = 1.83, *p* = 0.20; genotype × trial, *F*_(59,708)_ = 1.86, *p* = 0.0002). The mGluR5^KD-D1^ mice chose the alternative with the higher reward probability less frequently (Fig. 1*G*: genotype, *F*_(1,15)_ = 7.55, *p* = 0.015), and this difference depended on the trial number (genotype × trial, *F*_(59,885)_ = 1.43, *p* = 0.02), but to a lower extent than for the NR1^DATCreERT2^ mice. The choice behavior of the NR1^D1CreERT2^ mice did not differ from that of controls (Fig. 1*H*: genotype *F*_(1,13)_ = 3.32, *p* = 0.092; genotype × trial, *F*_(59,767)_ = 1.07, *p* = 0.34).

### Computational modeling results

We tested fits of three reinforcement learning models based on reward prediction error. Table 2 shows the LOOIC scores for the three models compared. For all groups tested, model 3 outperformed the others and had the lowest LOOIC scores by a large margin. Model 3 assumes that animals learn with different rates when the prediction error is positive or negative, and also that mice take the higher-order structure of the task into account, namely that they learn that at a given time only one of the ports gives high reward probability. Thus, in model 3 when unexpected reward is obtained following nose-poke to the left port, the expected reward associated with this port is increased, while the expected reward for the right port is decreased.

**Table 2.** Model comparisons using the LOOIC

Group	Model 1	Model 2	**Model 3**
NR1^DATCreERT2^ Control	16,167.7 9545.9	14,203.5 8304.4	**14,129.5** **8256.2**
mGluR5^KD-D1^ Control	18,640.8 14,216.2	17,888.8 12,743.5	**17,643.2** **12,557.1**
NR1^D1CreERT2^ Control	17,247.8 10,449.9	16,011.6 8753.4	**15,801.0** **8687.3**

Lower values of LOOIC indicate better model fits. The best performing model is highlighted with bold type; model 3 outperformed other models in all groups. Model 1, Separate learning rates for positive and negative reward PE; model 2, a single learning rate for PE and fictitious updating for the unchosen option; model 3, separate learning rates for positive and negative PE and fictitious updating for the unchosen option.

**Figure 2.**
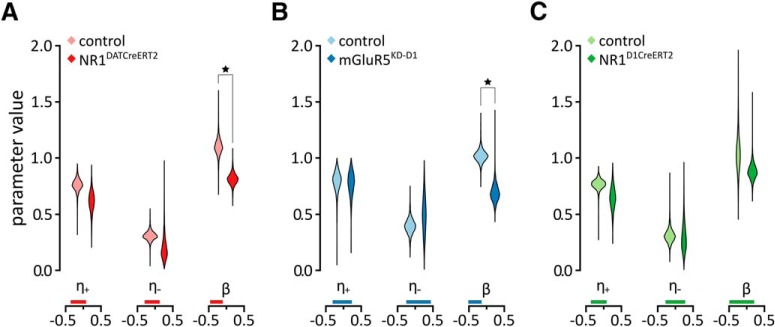
Computational modeling results. *A–C*, Density plots of posterior group parameter distributions with the best model (model 3) for the NR1^DATCreERT2^ (*A*), mGluR5^KD-D1^ (*B*), and NR1^D1CreERT2^ (*C*) strains. Credible differences are marked with stars, and vertical bars below the plots show 95% HDI ranges.

A summary of parameters calculated for the best-fitting model is shown in Figure 2*A*–*C*. For each parameter, we quantified an effect of the mutation by calculating the difference of hyperposterior distributions between mutant and control mice (Ahn et al., 2014), which is summarized as the 95% highest density interval (HDI). The 95% HDI refers to the range of parameter values that span the 95% of the distribution (Kruschke, 2014). If the 95% HDI of the difference is far >0 or <0, it indicates that there is a strong evidence of a group difference. While binary interpretations of 95% HDI should be avoided, it is possible to check whether the 95% HDI excludes 0 for a heuristic judgment of “credible” group differences. As in the case of previous analyses, credible effects of mutations were observed in the NR1^DATCreERT2^ mice (95% HDI = [−0.461, −0.102]) and mGluR5^KD-D1^ mice (95% HDI = [−0.516, −0.143]). We found that the mutation in the NR1^DATCreERT2^ and mGluR5^KD-D1^ strains affected the inverse temperature (*β*) parameter and mutant mice make more random rather than value-driven choices. However, the mutation did not cause a credible difference in the case of the NR1^D1CreERT2^ mice (95% HDI = [−0.492, 0.212]).

In agreement with the analysis of learning behavior of the NR1^DATCreERT2^ group (Fig. 1*F*), the means of posterior distribution of the learning rates for this group were lower than those of controls (Fig. 2*A*). However, unexpectedly, this effect was not credible (95% HDI = [−0.360, 0.078] for the reward learning rate; 95% HDI = [−0.295, 0.129] for the punishment learning rate). We did not observe any other credible effects of any of the mutations on learning rates. Another interesting observation was that in all groups, learning rates tend to be higher for positive than negative outcomes. Such a relationship between the learning rates has been observed before in a probabilistic choice task, and was proposed to arise because the animals might have learned that one option gives a higher reward on average, so a single reward omission may just be noise and should not change the behavior (Grogan et al., 2017). In summary, the computational modeling indicated that mutations significantly affected only the parameter influencing the preference for the alternative with a higher expected outcome. Additionally, the behavior in general was most consistent with models that included updates of the expected value of the nonselected alternative.

### Effects of prior outcomes on choice

**Figure 3.**
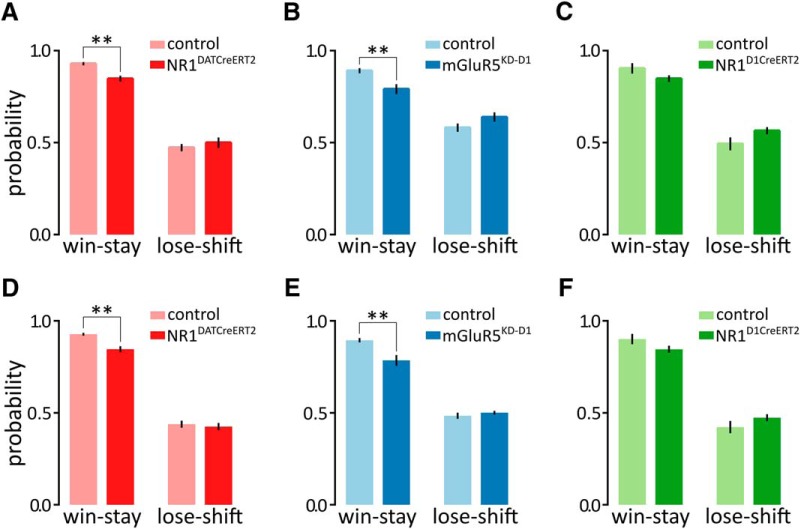
Effects of previous outcomes on choice. *A–C*, Probabilities of repeating the same choice when the previous response was rewarded (win-stay) or switching to an alternative choice when the preceding response yielded no reward (lose-shift) in the NR1^DATCreERT2^ (mutant, *n* = 6; control, *n* = 8; *A*), mGluR5^KD-D1^ (mutant, *n* = 8; control, *n* = 9; *B*), and NR1^D1CreERT2^ (mutant, *n* = 6; control, *n* = 9; *C*) strains. The probability of win-stay was calculated as the number of times the animal chose the same side as the side chosen during the previously rewarded trial divided by the total number of rewarded trials, while the lose-shift probability was calculated as the number of times the animal changed its choice when the preceding response yielded no reward divided by the total number of unrewarded trials. *D–F*, Simulation performance of the best model (model 3) with respect to mimicking win-stay/lose-shift choice behavior. Data are represented as the mean ± SEM. **p < 0.01 (*t* test).

To further assess the influence of previous outcomes on subsequent choices, we calculated the probabilities of repeating the same choice when the previous response was rewarded (win-stay) and switching to the alternative choice when the preceding response yielded no reward (lose-shift; Fig. 3*A*–*C*). The NR1^DATCreERT2^ and mGluR5^KD-D1^ mice were significantly less likely to repeat the previously rewarded choice than the control animals, whereas neither mutation affected the lose-shift ratio (Fig. 3*A*: win-stay, *t*_(12)_ = 4.059, *p* = 0.0016; lose-shift: *t*_(12)_ = 0.7093, *p* = 0.4917; Fig. 3*B*: win-stay, *t*_(15)_ = 3.159, *p* = 0.0065; lose-shift, *t*_(15)_ = 1.679, *p* = 0.1139). No significant effect of genotype on win-stay or lose-shift responding was observed in the NR1^D1CreERT2^ animals (Fig. 3*C*: win-stay, *t*_(13)_ = 1.72, *p* = 0.1091; lose-shift, *t*_(13)_ = 1.888, *p* = 0.0815).

The overall higher proportion of win-stay than lose-shift trials is in a qualitative agreement with the higher learning rate from positive than from negative feedback (Fig. 2). To test whether the model can quantitatively reproduce the proportions of win-stay trials and lose-shift trials, Figure 3*D*–*F* shows the simulation performance of model 3 with parameters set to the means of posterior distributions in Figure 2*A*–*C*. Comparisons of actual (Fig. 3*A*–*C*) and simulated (Fig. 3*D*–*F*) behavioral performance revealed that our model indeed describes observed data very well. Consistent with actual data, simulated NR1^DATCreERT2^ and mGluR5^KD-D1^ mice were significantly less likely to repeat the previously rewarded choice than the control animals (win-stay), but this was not observed in NR1^D1CreERT2^ simulated mice (Fig. 3*D*: win-stay, *t*_(12)_ = 3.939, *p* = 0.0020; Fig. 3*E*: win-stay, *t*_(15)_ = 3.292, *p* = 0.0049; Fig. 3*F*: win-stay, *t*_(13)_ = 1.657, *p* = 0.1215). We observed no effect of mutation on lose-shift behavior in any group, which is consistent with actual data (Fig. 3*D*: lose-shift, *t*_(12)_ = 0.4638, *p* = 0.6511; Fig. 3*E*: lose-shift, *t*_(15)_ = 0.8803, *p* = 0.3926; Fig. 3*F*: lose-shift, *t*_(13)_ = 1.432, *p* = 0.1757).

### Choice latency

**Figure 4.**
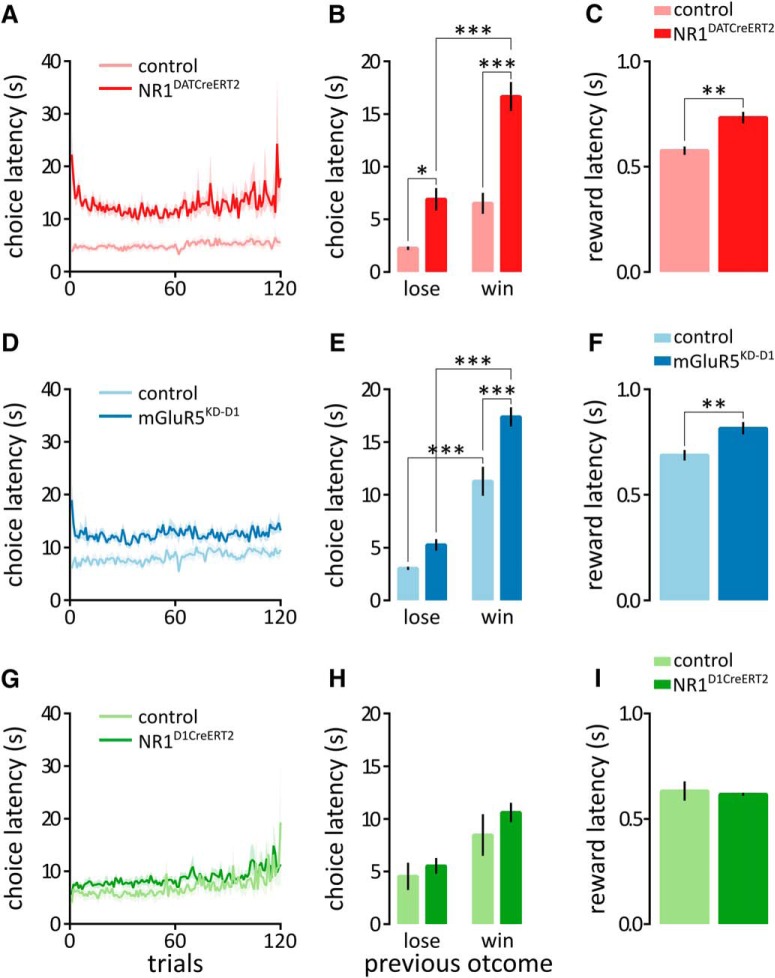
Reaction times in the probabilistic reinforcement learning task. *A–I*, Graphs show the reaction times observed in the NR1^DATCreERT2^ (mutant, *n* = 6; control, *n* = 8; *A–C*), mGluR5^KD-D1^ (mutant, *n* = 8; control, *n* = 9; *D–F*), and NR1^D1CreERT2^ (mutant, *n* = 6; control, *n* = 9; *G–I*) strains. *A*, *D*, and *G* show the time elapsed from the trial onset to the choice port entry. *B*, *E*, and *H* show the time from the new trial onset to the choice port entry following previously unrewarded (lose) or rewarded (win) trials. *C*, *F*, and *I* summarize the time from the reward delivery to the reward port entry. Values represent the mean choice latency (all sessions combined) ± SEM. *p < 0.05, **p < 0.01, ***p < 0.001 (Bonferroni-corrected *t* test or *t* test).

The analysis of the reaction times in the probabilistic reinforcement learning task revealed that the NR1^DATCreERT2^ and mGluR5^KD-D1^ mice required significantly more time to make a choice after the trial onset (Fig. 4*A*: genotype × trial, *F*_(119,1428)_ = 0.90, *p* = 0.7764; genotype, *F*_(1,12)_ = 34.89, *p* < 0.0001; trial, *F*_(119,1428)_ = 1.07, *p* = 0.2910; Fig. 4*D*: genotype × trial, *F*_(119,1785)_ = 0.84, *p* = 0.8871; genotype, *F*_(1,15)_ = 10.51, *p* = 0.0055; trial, *F*_(119,1785)_ = 3.62, *p* < 0.0001). Furthermore, the choice latency was strongly affected by the previous outcome, and the NR1^DATCreERT2^ and mGluR5^KD-D1^ mice spent more time choosing when the previous trial was rewarded (Fig. 4*B*: genotype × outcome, *F*_(1,24)_ = 6.15, *p* = 0.0205; genotype, *F*_(1,24)_ = 44.66; outcome, *F*_(1,24)_ = 40.23; both *p* < 0.0001; Fig. 4*E*: genotype × outcome, *F*_(1,30)_ = 5.04, *p* = 0.0323; genotype, *F*_(1,30)_ = 23.37; outcome, *F*_(1,30)_ = 139.21; both *p* < 0.0001). In addition, the NR1^DATCreERT2^ and mGluR5^KD-D1^ mice were slightly slower to collect their reward (Fig. 4*C*: *t*_(12)_ = 4.3, *p* = 0.0010; Fig. 3*F*: *t*_(15)_ = 3.242, *p* = 0.0055). Again, no effect of mutation on decision time or reward latency was observed in the NR1^D1CreERT2^ strain (Fig. 4*G*: genotype × trial, *F*_(119,1547)_ = 0.93, *p* = 0.6885; genotype, *F*_(1,13)_ = 1.45, *p* = 2499; trial, *F*_(119,1547)_ = 3.16, *p* < 0.0001; Fig. *4H*: genotype × outcome, *F*_(1,26)_ = 0.23, *p* = 0.6347; genotype, *F*_(1,26)_ = 1.70, *p* = 0.2043; outcome, *F*_(1,26)_ = 14.08, *p* = 0.0009; Fig. 3*I*: *t*_(13)_ = 0.4163, *p* = 0.6840). Therefore, the mutations in the NR1^DATCreERT2^ and mGluR5^KD-D1^ strains caused a delay in decision time.

### Reward magnitude discrimination and probability discounting

**Figure 5.**
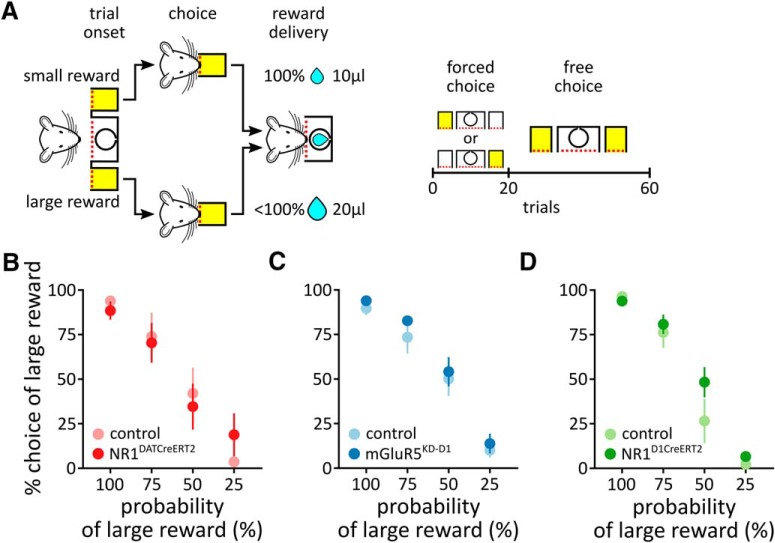
The probability-discounting task. *A*, Schematic representation of the probability-discounting task. One nose-poke port was associated with the delivery of small certain rewards, while the other nose-poke port was associated with the delivery of large uncertain rewards. Each session consisted of 20 forced trials during which only one port was active, followed by 40 free choice trials during which both ports were active. *B–D*, The graphs show the frequency of choosing the larger reward as a function of its probability in the NR1^DATCreERT2^ (mutant, *n* = 6; control, *n* = 7; *B*), mGluR5^KD-D1^ (mutant, *n* = 8; control, *n* = 9; *C*), and NR1^D1CreERT2^ (mutant, *n* = 5; control, *n* = 9; *D*) strains. Data are represented as the mean ± SEM.

**Figure 6.**
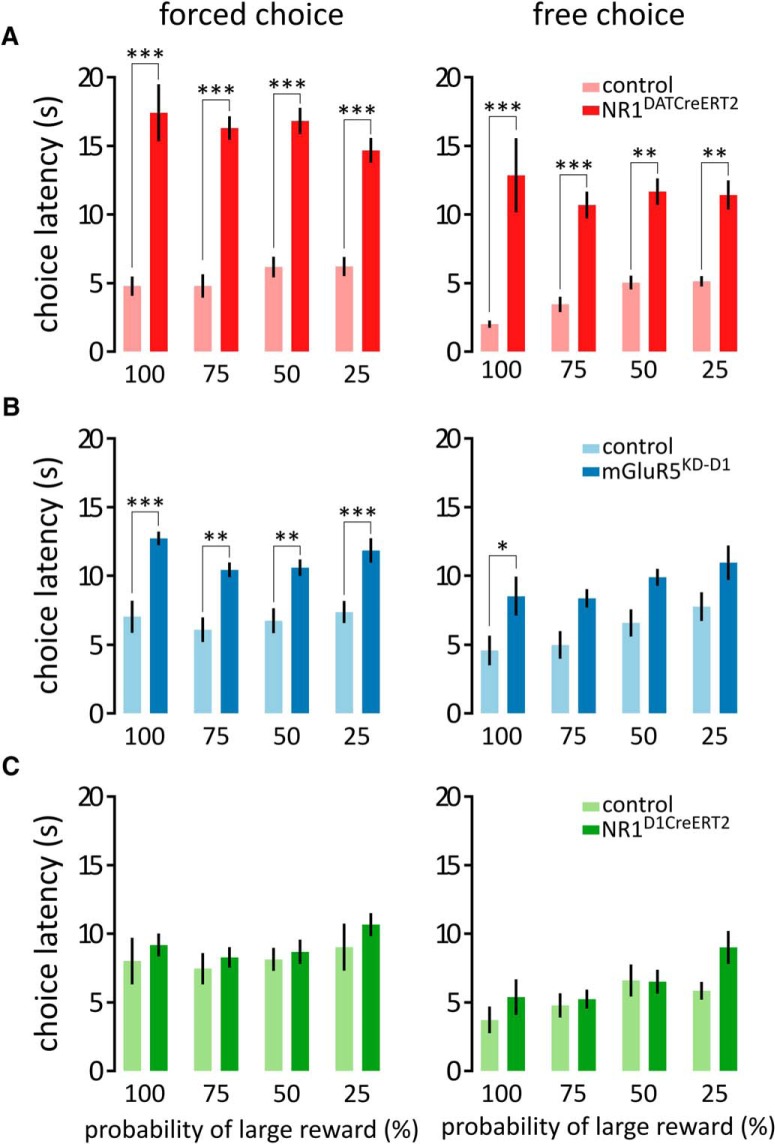
Reaction times in the probability-discounting task. *A–C*, Time elapsed from the trial onset to the choice port entry during the forced choice (left) and free choice (right) trials in the NR1^DATCreERT2^ (mutant, *n* = 6; control, *n* = 7; *A*), mGluR5^KD-D1^ (mutant, *n* = 8; control, *n* = 9; *B*), and NR1^D1CreERT2^ (mutant, *n* = 5; control, *n* = 9; *C*) strains. Bars represent the mean choice latency ± SEM. *p < 0.05, **p < 0.01, ***p < 0.001 (Bonferroni-corrected *t* test).

In the second experiment, we tested whether an ablation of glutamate receptors in the DA system influenced the discrimination of reward magnitude and discounting of the value of large outcomes caused by a decrease in the probability of large reward delivery. In this task, the animals were offered a choice between 10 or 20 μl of water (Fig. 5*A*). Each session began with 20 forced choice trials, during which the animals were familiarized with the choice outcomes, followed by 40 free choice trials. When both outcomes were deterministic and the animals were allowed to choose freely, the animals preferred the larger amount of water (5 d average ranged from 68.5% to 100%; mean, 92.6%; Fig. 5*B*–*D*). However, when the probability of receiving the larger reward gradually decreased, the preference for the large reward decreased accordingly, indicating that the animals perceived and adapted to the changes in the reward value (Fig. 5*B*: probability, *F*_(3,33)_= 39.53; Fig. 5*C*: probability, *F*_(3,45)_ = 109.92; Fig. 5*D*: probability, *F*_(3,36)_ = 109.92; all *p* < 0.0001). Although no effects of the mutations were observed on probability discounting (Fig. 5*B*: genotype × probability, *F*_(3,33)_ = 0.85, *p* = 0.4753; genotype, *F*_(1,11)_ = 0.0005, *p* = 0.9831; Fig. 5*C*: genotype × probability, *F*_(3,45)_ = 0.15, *p* = 0.9275; genotype, *F*_(1,15)_ = 0.67, *p* = 0.4250; Fig. 5*D*: genotype × probability, *F*_(3,36)_ = 1.77, *p* = 0.1706; genotype, *F*_(1,12)_ = 1.39, *p* = 0.2614), the analysis of the reaction times revealed a large increase in the latency to choose during both the forced choice and free choice trials in the NR1^DATCreERT2^ and mGluR5^KD-D1^ mice (Fig. 6*A*, forced choice: genotype × probability, *F*_(3,33)_= 3.11, *p* = 0.0396; genotype, *F*_(1,11)_ = 67.02, *p* < 0.0001; probability, *F*_(3,33)_ = 0.97, *p* = 0.4193; free choice: genotype × probability, *F*_(3,33)_= 1.81, *p* = 0.1642; genotype, *F*_(1,11)_ = 42.73, *p* < 0.001; probability, *F*_(3,33)_= 0.66, *p* = 0.5816; Fig. 6*B*, forced choice: genotype × probability, *F*_(3,45)_ = 1.42, *p* = 0.2486; genotype, *F*_(1,15)_ = 21.96, *p* = 0.0003; probability, *F*_(3,45)_ = 5.40, *p* = 0.0029; free choice: genotype × probability, *F*_(3,45)_ = 0.10, *p* = 0.9605; genotype, *F*_(1,15)_ = 9.14, *p* = 0.0085; probability, *F*_(3,45)_ = 6.11, *p* = 0.0014). This outcome was not observed in the case of the NR1^D1CreERT2^ mice (Fig. 6*C*, forced choice: genotype × probability, *F*_(3,36)_ = 0.71, *p* = 0.5533; genotype, *F*_(1,12)_ = 0.53, *p* = 0.4815; probability, *F*_(3,36)_ = 8.94, *p* = 0.0001; free choice: genotype × probability, *F*_(3,36)_ = 2.61, *p* = 0.0665; genotype, *F*_(1,12)_ = 0.88, *p* = 0.3673; probability, *F*_(3,36)_ = 9.09, *p* = 0.0001).

These results confirmed that while none of the mutations appreciably affected the magnitude discrimination or probability discounting, the animals from the NR1^DATCreERT2^ and mGluR5^KD-D1^ strains were considerably slower in performing choices.

## Discussion

The mutations in the NR1^DATCreERT2^ and mGluR5^KD-D1^ strains had three effects on the choice behavior. First, the performance in the probabilistic reinforcement learning task was impaired, leading to fewer choices of the alternative with the higher reward probability. This effect was transient in the NR1^DATCreERT2^ strain, and the mutant mice eventually reached the same performance as the controls, whereas the mGluR5^KD-D1^ animals showed a generally lower preference for the higher value option. Second, the NR1^DATCreERT2^ and mGluR5^KD-D1^ mice were less likely to repeat the previously rewarded choice. In accordance with this, computational modeling suggested that the behavior of both of these mutant groups was to a smaller extent influenced by acquired associations in comparison to controls (i.e., making more exploratory/random choices compared with controls). Finally, the third mutation effect in the NR1^DATCreERT2^ and mGluR5^KD-D1^ strains was an increase in the delay to make a choice. In contrast, there were no appreciable changes in the behavior of the NR1^D1CreERT2^ mice.

Earlier studies have shown that the inactivation of functional NMDA receptors in DA neurons impaired burst firing and attenuated phasic DA release in the striatum (Zweifel et al., 2009; Parker et al., 2010; Wang et al., 2011). Consistent with this finding, we recently reported that the induction of the mutation in the NR1^DATCreERT2^ mice causes a complete loss of NMDA receptor-dependent bursting of midbrain DA neurons (Jastrzębska et al., 2016). Considering the role of DA neuron burst firing in reward prediction error coding (Schultz et al., 1997; Glimcher, 2011), the observed effects of the mutation are to an extent unexpected, as no significant changes in learning rates were observed. Still, we note that the reduced win-stay probability is actually similar to the effect reported in the case of optogenetic studies, where the inhibition of DA neurons imitating negative reward prediction error reduced the likelihood of returning to the previously rewarded alternative (Hamid et al., 2016; Parker et al., 2016). Moreover, the study by Pessiglione et al. (2006) offers a possible explanation for why the reduced bursting of DA neurons might have led to less deterministic behavior rather than a reduced learning rate. In that study, the effects of a drug-reducing DA function on learning in an analogous task was studied in humans inside an fMRI scanner. The authors developed a computational model that captured both behavioral data and blood oxygenation level-dependent responses in striatum, which are known to correlate with reward prediction error. According to this model, the drug had an effect of reducing the value of reward parameter *r_t_* on trials where the reward is obtained (see Eqs. 1–4). Reducing *r_t_* has exactly the same effect on model behavior as reducing inverse temperature β (identified in our study for NR1^DATCreERT2^ and mGluR5^KD-D1^ mice) for the following reason. Reducing *r_t_* decreases the value to which the estimators *V_c,t_* converge, because they approach the expected value of the reward. If both *V*_1_*_,t_* and *V*_2_*_,t_* are reduced by the same constant, this constant can be taken outside the bracket in the softmax Equation 5 and incorporated into β giving a lower effective value of β. Computational models with reduced *r_t_* and β predict exactly the same behavior, and therefore cannot be distinguished on the basis of our data. Pessiglione et al. (2006) had additional neurophysiological data, indicating the value of reward prediction on each trial, which allowed them to distinguish between these models. Thus, in summary, the less deterministic behavior of NR1^DATCreERT2^ mice in our study might have resulted from impaired encoding of reward prediction error that led to reduced estimates of expected reward.

Nevertheless, we note that while the impairment caused by the mutation is clearly significant, it was arguably mild, and the NR1^DATCreERT2^ mice eventually reached the same performance as that observed in the control animals. This is in agreement with the observation that after extended training, performance levels in mice with constitutive mutations are similar to those found in control animals (Zweifel et al., 2009; James et al., 2015). Furthermore, in addition to its role in signaling reward prediction errors, phasic DA encodes expected reward value and contributes to risk-based decision-making (Fiorillo et al., 2003; Tobler et al., 2005; Sugam et al., 2012). This hypothesis is supported by observations in which the pharmacological blockade of DA receptors or the attenuation of phasic activity in DA neurons biases choices away from larger but probabilistic rewards (St Onge and Floresco, 2009; St Onge et al., 2011; Stopper et al., 2013, 2014). However, we found no effect of the loss of NMDA receptors on probability discounting, suggesting that NMDA receptors in DA neurons are not required for assessing the reward value when choosing between deterministic and probabilistic outcomes.

The inactivation of mGluR5 receptors in D_1_-expressing neurons decreased the frequency of choosing the alternative with a higher reward probability. Thus, the mGluR5^KD-D1^ mice made more random choices. Simultaneously, the NR1^D1CreERT2^ mice showed a normal performance. This result may be due to differences in the efficiency of the mutations in the dorsal part of the striatum. We have previously reported that a mutation in D_1_CreERT2-derived strains is efficient in the nucleus accumbens and ventral striatum but is less extensive in the dorsal parts of the striatum (Rodriguez Parkitna et al., 2010; Sikora et al., 2016), whereas, in the mGluR5^KD-D1^ strain, the mutation is efficient in both regions (Novak et al., 2010; Rodriguez Parkitna et al., 2013). The ventral components of the striatum are involved in stimulus-outcome learning, but the dorsal striatum plays a key role in learning about actions and their consequences (Balleine et al., 2007; Yin et al., 2008). A dissociable role of the ventral and dorsal striatal regions in choice behavior was also recently reported by Parker et al. (2016). These authors showed that DA terminals in the ventral striatum responded preferentially to reward consumption and reward-predicting cues, whereas terminals in the dorsal striatum responded more strongly to choices. Accordingly, optogenetic studies have demonstrated that the stimulation of D_1_ neurons in the dorsal striatum mimic changes in action values and bias choice behavior during decision-making (Tai et al., 2012). Therefore, we speculate that when glutamate receptor-dependent plasticity is disrupted at corticostriatal synapses in the dorsal, rather than the ventral striatum, an increased randomness in action selection occurs.

The strongest effect observed in our study was the increased delay in performing a choice in the NR1^DATCreERT2^ and mGluR5^KD-D1^ mice. This effect is consistent with a reported increase in the latency to choose in the appetitive T-maze task in NR1^DATCre^ mice (Zweifel et al., 2009) and the effect of optogenetic stimulation of DA neurons on the delay to engage in reward-seeking behavior (Hamid et al., 2016). Notably, our procedure imposed no limit on the trial length, while a 10 s limit was often used previously (Stopper et al., 2014; Parker et al., 2016). If a limit had been imposed, we would have likely observed a large number of omitted trials. Thus, a decision time limit could likely exacerbate the phenotypes observed in the probabilistic reinforcement learning task. It should also be noted that the mutations affected the time to collect the reward. However, only a slight increase in the reward latency was observed. The influence of the mutations on locomotor activity in this case seems to be rather unlikely. First, it was previously reported that a mutation in NR1^DATCreERT2^ mice had no effect on locomotor activity in the home cage or open field arena (Engblom et al., 2008), and only a mild reduction of activity in the novel environment was observed in mGluR5^KD-D1^ mice, with no change in the distance traveled in familiar environment (Rodriguez Parkitna et al., 2013). Second, based on the performance in the rotarod test, there is no evidence of motor impairment in NR1^DATCreERT2^ mice (Jastrzębska et al., 2016). We thus believe that an increase in choice latency is a result of an internal decision (or motivational) process, rather than a result of impaired motor performance. This interpretation is in line with observations showing that perturbations in mesolimbic DA signaling result in decreased motivation to engage in reward-seeking behavior, which is expressed as an increase in latency to initiate instrumental phase of reward-directed behavior (Nicola, 2010; Salamone and Correa, 2012).

In conclusion, we find that the loss of NMDA receptors in DA neurons and mGluR5 receptors in D_1_-expressing neurons affects the speed of the decision process and increases the number of exploratory choices. Nevertheless, mutant mice did improve their performance in the probabilistic reinforcement learning task and showed normal probability discounting. Overall, this indicates that reward-driven learning does occur in the absence of key receptors implicated in the plasticity of the reward system of the brain, but the decision-making process slows and loses efficiency.

## Synthesis

Reviewing Editor: Philippe Tobler, University of Zurich Decisions are customarily a result of the Reviewing Editor and the peer reviewers coming together and discussing their recommendations until a consensus is reached. When revisions are invited, a fact-based synthesis statement explaining their decision and outlining what is needed to prepare a revision will be listed below. The following reviewer(s) agreed to reveal their identity: Min Jung, Samantha Yohn. Note: If this manuscript was transferred from JNeurosci and a decision was made to accept the manuscript without peer review, a brief statement to this effect will instead be what is listed below.

Examining choice behavior of genetically modified mice in which glutamate receptors are deleted in dopamine-producing and dopamine-sensitive neurons provides new insights on the role of glutamate signaling in the brain's reward system.

The manuscript has been improved substantially from the previous version. Most of the concerns have been adequately addressed. In particular, the reinforcement learning model-based analysis yielded interesting results.

Major concerns:

Model 2 has two free parameters whereas models 1 and 3 have three free parameters. Accordingly, model 3 clearly fits the data better than model 1. However, is the additional free parameter of model 3 compared to model 2 justified? The authors may want to use AIC or BIC to compare their models and possibly compare also to an even simpler/baseline model (without learning at all), which may not be capable of simulating the strain differences.

The authors chose the 95% highest density interval to compare mutant and control groups. The authors should illustrate the interval graphically and compare parameter estimates (coefficients) between mutant and control groups.

The authors suggest that their results are due to actions in the dorsal striatum (i.e., we speculate that when glutamate receptor-dependent plasticity at corticostriatal synapses in the dorsal, rather than the ventral, striatum is disrupted, an increase in action selection occurs). However, mGluR5KD-D1 is expressed equally in both ventral and dorsal striatal subregions, as also noted by the authors. Please specify in the manuscript how these two statements can be reconciled?

The DATCreERT2 and mGluR5KD-D1 strains in general seem to produce very similar patterns of behavioral responding. However, Figure 2 seems to suggest that the magnitude of the effect between control and DATCreERT2 is larger compared to the effect seen with mGluR5KD-D1 and controls. The authors do not allude to why there are such distinct differences in these effects; if they are statistically significant, it will be important to highlight these differences in order to better understand the circuitry driving these behaviors.

The authors note that there may be an issue with the speed of the decision making process. Substantial work from Salamone and colleagues (i.e., Cousins et al. 1993; Yohn et al., 2015; Salamone and Correa, 2012) found a distinction in the limbic interface of motor/motivation. If a motor (i.e., speed) impairment is affecting choice, the results may be confounded. In Figure 2, the authors depict the latency and there is a clear difference in latency between genotypes tested. Is there a correlation between latency and choice impairment in the present data? Can the authors control for latency differences in the analyses? The authors did not put a latency cap on the time that needed to pass to have an omitted trial; however, was there a ceiling effect observed with NR1DATCreERT2 mice?

Additionally, while the authors note that there are no differences in locomotor activity between mutant strains and controls, is there a difference in intrinsic reinforcing properties of the reward. The authors should test to make sure the reinforcer is equally reinforcing between mutant and control animals, for example by performing a free feeding assay (Nunes et al. 2013; Yohn et al 2016) or providing other evidence for similar reward intake, independent of work output. If there is a reduction in one of the strains, it would indicate that either this reward is not as palatable for this strain as it is compared to the other strains or they satiate more quickly.

It will also be key to make sure there is not an attentional impairment between genotypes that is underlying the significant difference observed in percent of choosing the larger reward (i.e., visual tracking task). Perhaps these animals just have impairments encoding the cue that underlies the operant assay; was cue reactivity examined? The authors state that they did not look at omitted trials but doing so would inform this issue.

## Author Response

Dear Editor and Reviewers,

We thank you for the constructive comments on our manuscript and for being willing to consider publishing the manuscript in eNeuro. We have modified Figure 2 and revised the text in agreement with the suggestions. A point-by-point response to the comments can be found below. We feel that the manuscript has improved considerably and we hope that you now will find it suitable for publication.

Detailed responses:

Model 2 has two free parameters whereas models 1 and 3 have three free parameters. Accordingly, model 3 clearly fits the data better than model 1. However, is the additional free parameter of model 3 compared to model 2 justified? The authors may want to use AIC or BIC to compare their models and possibly compare also to an even simpler/baseline model (without learning at all), which may not be capable of simulating the strain differences.

We have updated Methods section to explain in more detail how leave-one-out cross validation information criterion (LOOIC) is computed, and explained its advantages over other methods (lines 208-215). An advantage of LOOIC approach is that model complexity (e.g., the number of model parameters) is naturally penalized in a Bayesian framework, as an overly complicated model will perform poorly on unseen data than a simpler model, and LOOIC directly assesses overfitting (Vehtari et al., 2017). Furthermore, we note that LOOIC is asymptotically equivalent to Akaike information criterion (AIC; Stone, 1977). However, unlike AIC or BIC, LOOIC is a fully Bayesian model comparison method, so we feel that using LOOIC is a more appropriate choice among multiple model comparison methods (i.e., instead of AIC or BIC).

We also feel it is unnecessary to compare our models against a model without learning. Simply because a model without learning would choose both alternatives with 50% probability throughout the experiment and would not replicate the pattern of behavior seen in Figure 1 (gradual learning over sessions) and Figure 3 (win-stay/lose-shift).

The authors chose the 95% highest density interval to compare mutant and control groups. The authors should illustrate the interval graphically and compare parameter estimates (coefficients) between mutant and control groups.

Figure 2 was updated and now includes a graphical representation of HDI intervals. Table 1 (statistical table) was also modified and includes 95% HDI intervals for all comparisons.

The authors suggest that their results are due to actions in the dorsal striatum (i.e., we speculate that when glutamate receptor-dependent plasticity at corticostriatal synapses in the dorsal, rather than the ventral, striatum is disrupted, an increase in action selection occurs). However, mGluR5KD-D1 is expressed equally in both ventral and dorsal striatal subregions, as also noted by the authors. Please specify in the manuscript how these two statements can be reconciled?

We believe there might be a misunderstanding. The discussion addresses differences between NR1D1CreERT2 and mGluR5KD-D1 strains (not NR1DATCreERT2). As indicated in the manuscript, we find the lack of the effect of the mutation in NR1D1CreERT2 mice surprising, bearing in mind the role of NMDA receptor dependent signaling in dorsal striatum in instrumental learning (Yin et al., 2005). We reasoned that if mutation in this strain is less effective in the dorsal striatum, this might explained the lack of the effect. On the other hand, since mutation in mGluR5KD-D1 mice was equally efficient in both striatal regions we speculatively suggested that glutamate receptor-dependent plasticity at corticostriatal synapses in the dorsal, rather than the ventral striatum may contribute to the observed phenotype. Our line of reasoning is based on the recent findings showing the role of dorsal striatum in encoding and biasing adaptive choice (Tai et al., 2012; Parker et al., 2016).

We made minor edits to the corresponding paragraph of the Discussion to make it more clear (lines 412-429).

The DATCreERT2 and mGluR5KD-D1 strains in general seem to produce very similar patterns of behavioral responding. However, Figure 2 seems to suggest that the magnitude of the effect between control and DATCreERT2 is larger compared to the effect seen with mGluR5KD-D1 and controls. The authors do not allude to why there are such distinct differences in these effects; if they are statistically significant, it will be important to highlight these differences in order to better understand the circuitry driving these behaviors.

In the manuscript we did not report comparisons between strains. In case of Figure 2, there are no credible differences between NR1DATCreERT2 and mGluR5KD-D1 mutant groups (95% HDI: -0.0668, 0.2855) or respective control groups (95% HDI: -0.1164, 0.2604). Mean values of the β parameter suggest the effect of the mutation is relatively similar (listed below).

control mutant difference

NR1DATCreERT2 1.10 0.817 -0.283

mGluR5KD-D1 1.02 0.698 -0,322

The authors note that there may be an issue with the speed of the decision making process. Substantial work from Salamone and colleagues (i.e., Cousins et al. 1993; Yohn et al., 2015; Salamone and Correa, 2012) found a distinction in the limbic interface of motor/motivation. If a motor (i.e., speed) impairment is affecting choice, the results may be confounded. In Figure 2, the authors depict the latency and there is a clear difference in latency between genotypes tested. Is there a correlation between latency and choice impairment in the present data? Can the authors control for latency differences in the analyses?

This is an interesting problem. In order to assess the effect of decision delay on outcome we performed a logistic regression (using the glm function in R) using time and previous outcome as factors. As a result we got a log odds ratio effect for each of the factors, which are plotted below:

In the figure, the dots represent log odds ratio of the effect of time (per second of delay till decision), and the bars show median values. From the figure it appears that the effect of time may be present if previous choice was rewarded (win), but please note that previous outcome strongly affects the delay to next decision (Figure 4 in the manuscript). The analysis suggests that longer intervals may be associated with lower probability of stay choices (negative log odds ratio), and this effect is possibly weaker in some mutants. However, again the NR1DATCreERT2 and mGluR5KD-D1 mutations were correlated with significantly longer delays to decision, thus the net effect could be the same irrespective of the genotype. Therefore, although the idea clearly merits further investigation, current data does not suggest that the delay is a factor driving the effects of the mutation. Also, please consider the results shown in Figure 5 in the manuscript mutants might be much slower, but it does not affect probability discounting.

The authors did not put a latency cap on the time that needed to pass to have an omitted trial; however, was there a ceiling effect observed with NR1DATCreERT2 mice?

With regard to a ceiling effect, we do not think there is one. Below are graphs showing probability density of interval length in NR1DATCreERT2 mice:

As shown in the figure, the distribution of delays to decision is shifted to the right in the mutant, but the shape of the distribution seems regular.

Additionally, while the authors note that there are no differences in locomotor activity between mutant strains and controls, is there a difference in intrinsic reinforcing properties of the reward. The authors should test to make sure the reinforcer is equally reinforcing between mutant and control animals, for example by performing a free feeding assay (Nunes et al. 2013; Yohn et al 2016) or providing other evidence for similar reward intake, independent of work output. If there is a reduction in one of the strains, it would indicate that either this reward is not as palatable for this strain as it is compared to the other strains or they satiate more quickly.

We do not believe that reward palatability or satiation level significantly contributes to the observed phenotype. Please note that the primary driver for responding is thirst (animals have limited access to water in the home cage). If satiety played a role it should be apparent in increased trial lengths and task disengagement in the final stages of the session. Based on the data in Figure 4 (panels A, D and G) this was not a major factor. Moreover, it is worth to note that all groups have had an opportunity to freely choose and consume reinforcer during the first phase of probability discounting task. In this task, animals showed the same level of initial reward preference, indicating that reinforcer was equally reinforcing.

In addition, we have previously reported that mutant mice from NR1DATCreERT2 strain showed normal fluid intake and preference for a saccharine solution in a 24h two-bottle choice test (Jastrzebska et al., 2016). This effect was replicated in an IntelliCage system in which fluid intake was measured over several days (ibid). Similarly, no effect of the mutation on water intake was observed in case of mGluR5KD-D1 mice (Rodriguez Parkitna et al., 2013). This further confirms that in either case, mutation had no effect on performance in free feeding assays.

We do acknowledge that the mutations may actually be affecting the value attributed to rewards, similarly to the effects reported in humans after treatment with L-DOPA or haloperidol (Pessiglione et al., 2006). In the revised Discussion we have introduced an extend fragment considering the possibility whether the observed effect of the mutations on the β parameter may actually reflect a change in reward value (lines 381-398).

It will also be key to make sure there is not an attentional impairment between genotypes that is underlying the significant difference observed in percent of choosing the larger reward (i.e., visual tracking task). Perhaps these animals just have impairments encoding the cue that underlies the operant assay; was cue reactivity examined? The authors state that they did not look at omitted trials but doing so would inform this issue.

We disagree with the notion that the observed phenotypes could be associated with attentional impairment or altered cue reactivity. First, animals underwent instrumental training, where in subsequent phases they learned to respond for cue-light (active nose-poke port) to obtain reinforcement, and all of them passed the training criteria. Second, all animals correctly responded for randomly presented cue-light during the forced-trial phase of the probability discounting task, even if during the free-choice phase they showed preference for one side. This indicates that cue reactivity and attention was not impaired.

As noted in previous responses, there were no omitted trials as experimental procedures imposed no limit on trial length. The only exception when trials were not analyzed was a situation in which no choice had been made before the end of the session (60 min had elapsed). This happened in case of one animal who ended 5th and 14th session of the probabilistic reinforcement learning task with 106 and 113 completed trials, respectively. In all other cases a complete 120 trials were included in analysis.

References:

Jastrzebska K, Walczak M, Cieslak PE, Szumiec L, Turbasa M, Engblom D, Blasiak T, Rodriguez Parkitna J (2016) Loss of NMDA receptors in dopamine neurons leads to the development of affective disorder-like symptoms in mice. Sci Rep 6:37171.

Parker NF, Cameron CM, Taliaferro JP, Lee J, Choi JY, Davidson TJ, Daw ND, Witten IB (2016) Reward and choice encoding in terminals of midbrain dopamine neurons depends on striatal target. Nat Neurosci 19:845854.

Pessiglione M, Seymour B, Flandin G, Dolan RJ, Frith CD (2006) Dopamine-dependent prediction errors underpin reward-seeking behaviour in humans. Nature 442:1042-1045.

Rodriguez Parkitna J, Sikora M, Golda S, Golembiowska K, Bystrowska B, Engblom D, Bilbao A, Przewlocki R (2013) Novelty-seeking behaviors and the escalation of alcohol drinking after abstinence in mice are controlled by metabotropic glutamate receptor 5 on neurons expressing dopamine d1 receptors. Biol Psychiatry 73:263270.

Stone M. (1977) An asymptotic equivalence of choice of model by cross-validation and Akaikes criterion. Journal of the Royal Statistical Society Series B. 39, 447.

Tai L-H, Lee AM, Benavidez N, Bonci A, Wilbrecht L (2012) Transient stimulation of distinct subpopulations of striatal neurons mimics changes in action value. Nat Neurosci 15:12811289.

Vehtari, A., Gelman, A., & Gabry, J. (2017). Practical Bayesian model evaluation using leave-one-out cross-validation and WAIC. Statistics and Computing, 27(5), 1413-1432.

Yin HH, Knowlton BJ, Balleine BW (2005) Blockade of NMDA receptors in the dorsomedial striatum prevents action-outcome learning in instrumental conditioning. Eur J Neurosci 22:505512.
